# Spontaneous subarachnoid hemorrhage: clinical impact, prognostic value and complications

**DOI:** 10.1186/cc9746

**Published:** 2011-03-11

**Authors:** M Mourelo-Fariña, A Aller-Fernández, P Vidal-Cortés, R Galeiras, M García

**Affiliations:** 1 University Hospital A Coruña, Spain

## Introduction

The aim of this study is to identify the characteristics of patients with spontaneous subarachnoid hemorrhage (SAH) and to analyze the complications, treatment, potential risk factors and prognostic value associated.

## Methods

A retrospective observational study of all patients admitted to our hospital with SAH during 4 years (2006 to 2009). We evaluate the functional outcome using the Glasgow Outcome Scale (GOS) at discharge and 6 months later. We compare variables with the chi-square and Student's *t *tests. Multiple regression analysis was performed.

## Results

A total of 168 patients were included: age 57.5 years (SD 14.9), 62.5% women, APACHE II 12 (SD 6.7), Glasgow Coma Scale (GCS) 9.9 (SD 6.5). Punctuation in clinical grading scales was: Hunt-Hess (H-H) 2.8 (SD 1.5); Fisher 3.0 (SD 1.0); World Federation Neurosurgeons Scale (WFNS) 2.8 (SD 1.5). Personal antecedents: arterial hypertension (32.1%) followed by drug use (31.2%). Presentation was headache in 62.1%. We perform CT angiography in 9.6% and arteriography in 78.6% (delay was 1 day). We found no aneurysm in 24.6%. The embolization was complete in 63.4%. The localization of the aneurysm was more frequent in the anterior communicating artery. Surgical treatment was performed in 2.2%. Complications of SAH: vasospasm 31.5% (managed with triple-H therapy 71.7%), ischemic stroke occurred in 60.4%; 4.2% rebleeding; hydrocephalus in 23.2%. Mortality risk factors: univariate analysis found age (*P *= 0.004), worsening control CT (*P *< 0.01), rebleeding (*P *< 0.01), coma (*P *= 0.02), hydrocephalus (*P *< 0.01), intracranial hypertension (*P *= 0.002), H-H (*P *< 0.01), Fisher (*P *< 0.01), WFNS (*P *< 0.01), initial GCS (*P *< 0.01), GOS at discharge to ICU (*P *= 0.002) and time to embolization (*P *= 0.02). Multivariate analysis predictors of mortality: GCS at admission and at discharge to ICU (*P *= 0.013), worsening in control CT (*P *= 0.004) and length of stay (LOS) in the ICU (*P *= 0.04). ICU LOS was 10.6 days (SD 9.9) and hospital LOS was 56.7 days (SD 26.3). Global ICU mortality was 29.2% (77.5% brain death).

## Conclusions

The most frequent complications found were ischemic stroke, vasospasm and hydrocephalus. In our study we found that clinical grading scales predict mortality in univariate analysis. Predictors of mortality in SAH were age, GCS at admission and discharge; control CT, delay to embolization, and complications related to SAH are strong mortality predictors. In most patients, death is related to SAH complications.
